# Association between diversity levels of member composition in group activities of older adults and the occurrence of need for care: the JAGES 2013–2019 longitudinal study

**DOI:** 10.1186/s12877-023-04261-x

**Published:** 2023-09-20

**Authors:** Nao Shimizu, Kazushige Ide, Katsunori Kondo

**Affiliations:** 1 Department of Physical Therapy, Faculty of Health Sciences, Ryotokuji University, 5-8-1 Akemi, Urayasu-Shi, Chiba, 279-8567 Japan; 2 Department of Public Health, Graduate School of Medicine, Chiba University, Yayoi-Cho, Inage, Chiba, 263-8522 Japan; 3 Center for Preventive Medical Sciences, Chiba University, Yayoi-Cho, Inage, Chiba, 263-8522 Japan; 4 Department of Community General Support, Hasegawa Hospital, Yachimata, Chiba 289-1113 Japan; 5 Center for Gerontology and Social Science, Research Institute, National Center for Geriatrics and Gerontology, 7-430 Morioka-Cho, Obu, Aichi 474-8511 Japan

**Keywords:** Older adult, Group participation, Member diversity, Longitudinal study, Long-term care

## Abstract

**Background:**

Participating in groups with diverse members is associated with improved health among older adults. The study examined the relationship between diversity of group members and needed support or long-term care.

**Methods:**

We conducted a longitudinal study for the Japan Gerontological Evaluation Study with 61,281 participants aged ≥ 65 years who were surveyed in 2013 and followed-up for six years. We assessed three dimensions of the diversity of the participating members (sex, age, and region of residence). We then graded the diversity level into four categories: level 0 (not in any group), level 1 (in a group without diversity or in a group with diversity in one of the three factors), level 2 (in a group with diversity in two of the three factors), or level 3 (in a group with diversity across all factors). We adjusted for 12 covariates using Cox hazard survival analysis models with hazard ratios (HRs) and 95% confidence intervals (CIs) estimated for the association between group members’ diversity levels and needed support or long-term care. The same study was conducted when stratified by employment status at baseline.

**Results:**

Participants in social participation groups with more diverse group members had a lower incidence of needed support or long-term care as compared to their counterparts. Compared to those with no participation group, HR decreased by 14% to 24% with increasing levels of diversity. The HR for the level of care needed for participants in the social participation group with high residential diversity was 0.89 (95% CI: 0.84–0.94). For participants who were currently unemployed, HR reductions ranged from 16%–28% with increasing levels of diversity compared to the non-participating group. No association was found for employed participants.

**Conclusions:**

The reason the HRs of Japanese elderly people certified as needing support or care are lower when the diversity of participating groups is higher could be owing to the presence of a variety of people and the diversification of social networks, which facilitates the building of bridging social relational capital. Public health policies that encourage participation in diverse organizations will be important in the future.

**Supplementary Information:**

The online version contains supplementary material available at 10.1186/s12877-023-04261-x.

## Background

The aging rate as a percentage of the world’s total population is projected to rise from 5.1% in 1950 to 9.3% in 2020 and 17.8% in 2060, making long-term care prevention a global priority [1]. One public health measure that can intervene in aging is the “Age-Friendly City” concept proposed by the World Health Organization (WHO) [2]. In particular, efforts to promote social participation, which constitutes “social capital,” are vital. In Japan, where the population is aging most rapidly, there is a need to establish and implement care prevention measures and evidence to promote social participation.

There are several studies on social participation and health. Among older adults who participate in community organizations, social participation is associated with lower risk [3] and hazard ratios (HRs) for mortality, onset of dementia [4], depression, motor function decline [5], decline in activities of daily living [6], and functional disability [7, 8].

The number and type of social participation have different associations with health [7, 9]. However, few studies have focused on the membership composition of the groups in which people participate.

Groups with diverse members improve social capital, and the more diverse the active group’s sex ratio, residential area, and age structure, the higher the subjective view of health [10, 11]. A longitudinal study by Iwase et al. found that participating in groups with diverse members is associated with improved health among older adults [12].

There are also many reports on the relationship between employment and health among older adults [13–15]. Employment among them is related to health as a form of social participation [14]. Retirement could sever social ties and affect health [13].

However, there are no reports on the risk of requiring long-term care and diversity of group membership, and there are insufficient longitudinal studies to clarify the causal relationships. Therefore, the current study purpose was to clarify the association between diversity of group membership and the occurrence of care and support needs certification among older adults. In addition, as a subgroup analysis, we examined the relationship with employment.

## Methods

### Data source

We used data from the Japan Gerontological Evaluation Study (JAGES), which surveyed health behaviors, psychological factors, and socioeconomic factors among older adults aged ≥ 65 years, who had not been certified as requiring assistance or care approximately every three years since 2013 [16, 17]. The study used cohort data from a six-year follow-up of respondents surveyed in 2013 in 30 municipalities.

### Participants

Self-report questionnaires were mailed to 192,231 functionally independent older adults aged ≥ 65 years (71.1% response rate); baseline survey was conducted in 2013; 73,262 participants were followed for six years. We excluded 7,511 individuals who were lost to follow-up and 4,470 individuals who had limited activities of daily living at baseline. The final analysis included 61,281 participants.

### Outcome

The objective variable was whether participants were certified as requiring support or higher level of care during the follow-up period, as in previous studies [18]. In case of death, moving out, and so on, the follow-up was terminated. Data on the certification of requiring long-term care by long-term care insurers, and on the imposition of long-term care insurance premiums (using information on the discontinuation of imposition owing to death, moving out, etc.) were used to determine the certification of requiring long-term care and death. The date of occurrence of all certifications and that of application for certification of long-term care required. To protect personal information, addresses and names were deleted, and the insured person’s number used for identification was encrypted by each insurer, so that the researcher could not identify the individual. The encrypted person’s number was used to match the questionnaire data with the data on the certification of long-term care needs and deaths.

### Explanatory variables

Explanatory variables were defined based on previous studies [11]. Group member diversity factors and group member diversity levels were used. Participants were asked if they participated in 14 types of groups: volunteer, sports, hobbies, senior citizen clubs, neighborhood associations, learning and culture, care prevention and health promotion, sharing special skills and experiences, community events, watching over older adults, support for older adult, childcare support, community environmental improvement, and other groups.

Respondents who indicated that they participated in these groups were further asked to rate the diversity of the members of the groups in which they participated most often. The diversity of the group members was rated in terms of sex, region of residence, and age structure, as defined below:

1. Sex: female or male only (not diverse); mixed (diverse)
2. Region of residence: only from the same municipality (not diverse); some from other municipalities (diverse)
3. Age mainly same generation (not diverse); mix of generations whose ages differ by more than 20 years (diverse)

Dummy variables were created for each dimension of diversity (sex, region of residence, and age).

Based on this information, participants were classified into four groups: level 0 (not in any group), level 1 (in a group without diversity or in a group with diversity in one of the three factors), level 2 (in a group with diversity in two of the three factors), or level 3 (in a group with diversity across all factors). This was defined as the diversity level (Table S1). We also created dummy variables for each dimension of the diversity (sex, residential area, and age).

### Covariates

We defined the covariates based on previous studies [11, 18–21], which were: sex (male, female), age (65–74, ≥ 75), education (< 9 years, 10–12 years, ≥ 13 years), equivalent income (< 1.5 million, 1.5–2.49 million, ≥ 2.5 million/year), current work status (employed, unemployed), marital status (married, never married, bereaved/divorced), population density (rural = less than 1000, suburban = 1000–3999, urban =  ≥ 4000), medical history (heart disease, cerebrovascular disease, cancer) frequency of seeing friends or acquaintances (at least once a week, less than once a week), frequency of going out (at least four times a week, less than four times a week).

### Statistical analysis

Multiple assignment methods were used for missing values and for all data including diversity levels and factors, and other covariates. Twenty datasets were created with missing values assigned, and Cox hazard regression analyses were estimated for each dataset and adjusted for all covariates; 95% confidence intervals (CIs) were estimated for the diversity levels of group activities and for the HR between the diversity factor and the need for care. Twenty HRs and 95% CIs from the datasets were combined into a single composite HR and 95% CI. Because of the diversity of group members and current work status interaction, participants were stratified by employment status at baseline and the same study was performed. STATA V.16 (Stata Corp, College Station, TX, USA) was used to conduct a statistical analysis, with a significance level of 5%.

As a sensitivity analysis, the same study was conducted using the complete data. In addition, we examined the association between diversity level (dimension) and needed support or long-term care for only one group of participants. We checked for multicollinearity on the group member diversity variable.

### Ethical considerations

Ethical approval for this study was obtained from the ethics committee of Chiba University (no. 3442) and Ryotokuji University (no. 22–19). This study was conducted in accordance with the principles of the Declaration of Helsinki. All participants were informed before this study was conducted that their participation was voluntary.

## Results

We analyzed data from 61,281 individuals who were traceable for six years starting in 2013. The following missing data were processed using multiple assignment methods: information on population or diversity dimensions (40,563), employment status (5,060), marital status (2,917), comorbidity status (8,667), equivalent income (8,939) and education (1,105). During the study period, 12,765 (20.8%) participants were certified as needed support or long-term care. Table 1 shows the characteristics of those who were certified as needed support or long-term care and those who were not. HRs and 95% CIs for the diversity of the participation group related to the presence or absence of certification of need for assistance or care are shown in Table 2. The incidence of needing assistance or care decreased from 14 to 24% as the diversity of group members increased, compared to personnel who did not participate in group activities. Diversity of group members’ residential areas significantly reduced the incidence of need for assistance and care certification (HR = 0.89; 95% CI; 0.84–0.94).

**Table 1:** Characteristics of participants who have been certified as needing long-term care and who have not been certified as needing long-term care

Characteristics	Long-term care certification, n(%)^a^		*P* value
	Yes	No	
total	12,765(20.8)	48,516(79.2)	
Diversity level^b^			
Level 0	5,427(42.5)	18,173(37.5)	< 0.001
Level 1	1,761(13.8)	6,092(12.5)	
Level 2	991(7.8)	5,653(11.7)	
Level 3	522(4.1)	3,438(7.1)	
Each dimension of diversity			
Gender	3,131(24.5)	15,895(32.8)	< 0.001
Residential area	1,307(10.2)	8,396(17.3)	< 0.001
Age composition	1,443(11.3)	8,435(17.4)	< 0.001
Age			
65 < 74	3,567(27.9)	33,599(69.3)	< 0.001
≥ 75	9,198(72.1)	14,917(30.7)	
sex			
Men	5,641(44.2)	23,118(47.7)	< 0.001
Women	7,124(55.8)	25,398(52.3)	
Current workers	1,335(10.5)	11,922(24.6)	< 0.001
Marital Status			
Married	7,746(60.7)	36,152(74.5)	< 0.001
Single	259(2.0)	1,034(2.1)	
Widowed/divorced	4,281(33.5)	10,101(20.8)	
Population density			
Rural	1,977(15.5)	7,630(15.7)	< 0.001
Suburbs	5,831(45.7)	24,851(51.2)	
City	4,957(38.8)	16,035(33.1)	
Comorbidity			
Cancer	619(4.8)	1546	< 0.001
Cardiac disease	1,864(14.6)	4,474(9.2)	< 0.001
Stroke	597(4.7)	1,219(2.5)	< 0.001
Income			
< 1.5 million yen	3,590(28.1)	11,158(23.0)	< 0.001
1.5–2.4 million yen	4,089(32.0)	17,613(36.3)	
≧2.5 million yen	2,563(20.1)	12,901(26.6)	
Education			
< 9 years	5,864(45.9)	17,998(37.1)	< 0.001
10–12 years	4,350(34.1)	19,416(40.0)	
≧13 years	2,179(17.1)	10,369(21.4)	
Frequency of meeting acquaintances(more than once a week)	5,591(43.8)	24,271(50.0)	< 0.001
Frequency of going out(more than once a week)	7,833(37.9)	37,636(77.6)	< 0.001

^a^The total numbers may vary across the characteristics because of missing data. The percentage may not total 100 because of rounding

^b^We assessed three dimensions of the diversity (sex, age, and region of residence). We then graded the diversity level into four categories: level 0 (not in any group), level 1 (in a group without diversity or in a group with diversity in one of the three factors), level 2 (in a group with diversity in two of the three factors), level 3 (in a group with diversity across all factors)

**Table 2:** Combined prevalence ratios and 95% confidence intervals for long-term care certification estimated by cox proportional hazard model with multiply imputed data

Characteristics	HR(95%CI)			
	Model crude	*P* value	Model1^a^	*P* value
Diversity level(range0-3)				
Level 0	reference		reference	
Level 1	0.84(0.79–0.89)	< 0.001	0.86(0.81–0.90)	< 0.001
Level 2	0.70(0.66–0.75)	< 0.001	0.80(0.75–0.86)	< 0.001
Level 3	0.62(0.57–0.67)	< 0.001	0.76(0.70–0.82)	< 0.001
Each dimension of diversity				
Gender	1.29(1.17–1.42)	< 0.001	1.10(0.99–1.22)	0.081
Residential area	0.77(0.73–0.82)	< 0.001	0.89(0.84–0.94)	< 0.001
Age composition	0.89(0.84–0.94)	< 0.001	0.98(0.93–1.04)	0.575
Age				
75 ~			4.18(4.02–4.36)	< 0.001
Women			1.00(0.96–1.04)	0.964
Current workers			0.64(0.60–0.67)	< 0.001
Marital Status				
Married			reference	
Single			1.33(1.28–1.39)	< 0.001
Widowed/divorced			1.26(1.06–1.49)	0.009
Population density				
Rural			reference	
Suburbs			0.84(0.81–0.87)	< 0.001
City			0.78(0.74–0.82)	< 0.001
Cancer			1.54(1.42–1.67)	< 0.001
Cardiac disease			1.30(1.28–1.39)	< 0.001
Stroke			1.51(1.39–1.64)	< 0.001
Income				
< 1.5 million yen			reference	
1.5–2.4 million yen			0.88(0.84–0.92)	< 0.001
≧2.5 million yen			0.84(0.79–0.88)	< 0.001
Education				
< 9 years			reference	
10–12 years			0.93(0.89–0.96)	< 0.001
≧13 years			0.93(0.88–0.98)	0.005
Frequency of meeting acquaintances(more than once a week)			0.87(1.11–1.20)	< 0.001
Frequency of going out(more than once a week)			0.54(0.84–0.90)	< 0.001

*CI* confidence interval

^a^Hazard ratios of the diversity level estimated by cox proportional hazard model, adjusted for sex, age, education, equivalent income, current work status, marital status, population density, medical history (heart disease, cerebrovascular disease, cancer) frequency of seeing friends or acquaintances, frequency of going out

Participants who were not currently employed had a 16%–28% (HR) reduction in the rate of care certification for each level of diversity compared to those who were currently unemployed (Table 3). For working participants, there was no association between needed support or long-term care and diversity of the participating group (Table 4).

**Table 3:** Combined prevalence and 95% confidence intervals for long-term care certification for those not working estimated by Cox proportional hazards models using multiple imputation data(*n* = 42,964)

Characteristics	HR(95%CI)			
	Model crude	*P* value	Model1^a^	*P* value
Diversity level(range0-3)				
Level 0	reference		reference	
Level 1	0.78(0.73–0.83)	< 0.001	0.84(0.79–0.90)	< 0.001
Level 2	0.68(0.63–0.73)	< 0.001	0.79(0.74–0.85)	< 0.001
Level 3	0.60(0.54–0.65)	< 0.001	0.72(0.65–0.79)	< 0.001
Each dimension of diversity				
Gender	1.30(1.16–1.46)	< 0.001	1.09(0.97–1.23)	0.142
Residential area	0.78(0.74–0.83)	< 0.001	0.88(0.83–0.93)	< 0.001
Age composition	0.93(0.80–0.99)	0.030	0.99(0.93–1.04)	0.605
Age				
75 ~			4.08(3.90–4.27)	< 0.001
Women			0.99(0.95–1.04)	0.788
Marital Status				
Married			reference	
Single			1.31(0.87–0.96)	< 0.001
Widowed/divorced			1.27(1.04–1.56)	0.001
Population density				
Rural			reference	
Suburbs			0.83(0.80–0.87)	< 0.001
City			0.77(0.73–0.82)	< 0.001
Cancer			1.55(1.42–1.70)	< 0.001
Cardiac disease			1.32(1.24–1.39)	< 0.001
Stroke			1.51(1.37–1.65)	< 0.001
Income				
< 1.5 million yen			reference	
1.5–2.4 million yen			0.87(0.83–0.92)	< 0.001
≧2.5 million yen			0.84(0.79–0.89)	< 0.001
Education				
< 9 years			reference	
10–12 years			0.91(0.87–0.95)	< 0.001
≧13 years			0.91(0.85–0.96)	0.001
Frequency of meeting acquaintances(more than once a week)			0.55(0.50–0.60)	< 0.001
Frequency of going out(more than once a week)			0.87(0.84–0.90)	< 0.001

*CI* confidence interval

^a^Hazard ratios of the diversity level estimated by cox proportional hazard model, adjusted for sex, age, education, equivalent income, current work status, marital status, population density, medical history (heart disease, cerebrovascular disease, cancer) frequency of seeing friends or acquaintances, frequency of going out

**Table 4:** Combined prevalence and 95% confidence intervals for long-term care certification for those who work, estimated by Cox proportional hazards model with multiple imputation data(*n* = 13,257)

Characteristics	HR(95%CI)			
	Model crude	*P* value	Model1^a^	*P* value
Diversity level(range0-3)				
Level 0	reference		reference	
Level 1	0.89(0.71–1.10)	0.273	0.89(0.72–1.10)	0.277
Level 2	0.87(0.72–1.05)	0.031	0.87(0.72–1.05)	0.159
Level 3	0.97(0.79–1.19)	0.131	0.96(0.79–1.19)	0.74
Each dimension of diversity				
Gender	1.25(0.95–1.65)	0.114	1.10(0.85–1.42)	0.489
Residential area	0.86(0.76–0.97)	0.017	0.95(0.83–1.08)	0.426
Age composition	0.95(0.82–1.10)	0.503	1.00(0.86–1.16)	0.998
Age				
75 ~			4.90(4.38–5.47)	< 0.001
Women			1.07(0.95–1.21)	0.263
Marital Status				
Married			reference	
Single			1.30(1.38–1.48)	< 0.001
Widowed/divorced			1.37(0.84–2.25)	0.005
Population density				
Rural			reference	
Suburbs			0.85(0.76–0.96)	0.010
City			0.76(0.65–0.91)	0.002
Cancer			1.53(1.18–1.98)	< 0.001
Cardiac disease			1.36(1.15–1.60)	< 0.001
Stroke			1.72(1.30–2.28)	< 0.001
Income				
< 1.5 million yen			reference	
1.5–2.4 million yen			0.90(0.78–1.04)	0.147
≧2.5 million yen			0.81(0.70–0.94)	0.005
Education				
< 9 years			reference	
10–12 years			0.90(0.80–1.03)	0.121
≧13 years			1.01(0.87–1.18)	0.890
Frequency of meeting acquaintances(more than once a week)			0.37(0.29–0.47)	< 0.001
Frequency of going out(more than once a week)			0.89(0.79–1.00	0.041

*CI* confidence interval

^a^Hazard ratios of the diversity level estimated by cox proportional hazard model, adjusted for sex, age, education, equivalent income, current work status, marital status, population density, medical history (heart disease, cerebrovascular disease, cancer) frequency of seeing friends or acquaintances, frequency of going out

The results of the sensitivity analysis with complete data were identical to the result with multiple imputation. Sensitivity analysis showed similar results in the complete data. When examined in participants who participated in only one group, the HR for the occurrence of needed support or long-term care was reduced by participation in groups with two or more diversity dimensions.

Multicollinearity was examined, and no differences in HR in the survival analysis were found (Tables S2, S3 and S4). Correlations between variables were also examined, with a correlation coefficient of 0.14 for age diversity and residential area diversity of group members, 0.04 for sex diversity and residential area diversity, and 0.01 for age diversity and sex diversity (Table S5).

## Discussion

Among Japanese seniors, the greater the diversity of the members of the organizations in which they participate, the lower the rate of certification of support and care needed by the participating members. This result was similar to that of a previous cross-sectional study in which the outcome was a subjective sense of health [11].

The association between the occurrence of care needs in older adults and the diversity of participating groups could be related to the diversity of social networks established by group participants. Social networks are associated with physical and mental health, decreased depression [22], and maintenance of self-efficacy [23]. Social networks reinforce social roles, foster group values and attachments, and promote social participation and involvement [24, 25]. They are also said to stimulate individual creativity, increase flexibility of thought, and improve decision-making [26]. For older adults, who often experience negative events such as illness, partner separation, or unemployment, there is concern that reduced self-efficacy and loss of social roles can lead to a shrinking community and social isolation. Social isolation can negatively impact health (including physical ailments) [27], increase mortality [28], and cause depression [29]. The diversity of social networks creates new interpersonal relationships and participation in new communities beyond the participating organizations. Social network diversity promotes increased physical activity [30]. We believe that the increase in new interpersonal relationships and social activities could have impacted health by increasing the self-efficacy and sense of purpose in life of older adults.

We also believe that the diversification of individuals’ social networks and participation in new communities led to better access to health-related material resources and increased health activity, leading to the current results [31, 32]. Social participation increases the amount of physical activity, and enhanced social networks facilitate access to social resources such as health-related information and places that provide it, leading to improved cognitive and physical function and reduced mortality [33]. Leisure activities are associated with health [34, 35]. The decline in mobility associated with aging tends to limit the range of activities and makes people more likely to engage in unconventional behaviors. By interacting with people of different ages, sexes, and residential areas, people could learn about new nearby medical facilities and health-related events. In addition, given the sex and age bias among participants in some leisure activities [35], the exchange of information within the same group could have triggered participation in new leisure activities.

As reported by Zaitsu et al. [11] and Aida et al. [33], diversity of activity groups is a factor that promotes the building of bridging social capital within an activity group. Connections among people with common characteristics are defined as bonding social capital, while connections among people with different social characteristics are defined as bridging social capital [36, 37]. Bridging social capital enables access to resources outside of the immediate social environment and is associated with health [12]. As described above, the presence of diverse people and the diversification of social networks could have facilitated the construction of bridging social capital.

The results differed depending on current employment status. No significant differences were found among those who were employed; only among those who were unemployed, diversity of participating groups was effective in reducing the number of people requiring assistance or care. There is an association between retirement and health among older adults [38, 39], and working older adults are healthier than those who are unemployed [40]. Among older adults, those who work have more opportunities to interact with a diverse group of people at work, and we believe that the impact of the diversity of people they interact with at work is greater than that of those who interact with a diverse group of people in community activities, in which they participate less frequently.

There were limitations to this survey. First, because the diversity of the most frequently attended groups was analyzed, it was not possible to investigate the diversity of the multiple participation groups. Older adults who participate in multiple groups can benefit more from multiple participation than older adults who participate in only one group [7, 41]. To address this issue, a sensitivity analysis was conducted with those who participated in only one group. Those who participated in groups with diverse members had lower rates of care needs than those who participated in non-diverse groups. Thus, this limitation could not affect the conclusion. Second, there were many missing values. In response, we calculated missing values that included all variables by making multiple substitutions, but this is a concern. A sensitivity analysis was performed on the complete data to address this issue and the results were identical to the final results from multiple imputation. The discussion of negative aspects of social capital was incomplete. One negative aspect of social capital is that organizations with strong networks create exclusivity, which could affect health [42]. This point requires further investigation. The definition of the diversity level variable differs from previous studies. In a previous study [11], diversity level was defined as a continuous variable with five groups (0–4), while in this study it was defined as a categorical variable with four groups (0–3). The reason is that the Kaplan–Meier method was used to validate the results, and crossings were observed in the two groups. Therefore, the two groups were combined and treated as one group. We believe this is a problem of the number of groups, but further study is needed.

The higher the diversity of group members, the lower the incidence of needing assistance and care, and that diversity of residence is a more relevant and important dimension. Further research is needed to clarify the relationship between group diversity and the perception of requiring support and care in older adults’ population.

### Supplementary Information

**Additional file 1: Table S1. **Details of group member diversity level variables.

**Additional file 2: Table S2. **Combined prevalence ratios and 95% confidence intervals for long-term care estimated by cox proportional hazard model with complete data.

**Additional file 3: Table S3. **Combined prevalence and 95% confidence intervals for long-term care certification for those who work, estimated by Cox proportional hazards model with complete data.

**Additional file 4: Table S4. **Combined prevalence and 95% confidence intervals for long-term care certification for those not working , estimated by Cox proportional hazards model with complete data.

**Additional file 5: Table S5. **Combined prevalence and 95% confidence intervals of long-term care estimated by Cox proportional hazards models with multiple imputation data (subjects in one participation group only,*n*=11,921).

## Data Availability

Data are available upon reasonable request. The JAGES datasets, which were used in this research, are available from the corresponding author upon reasonable request. All inquiries should be addressed to the data management committee via e-mail: dataadmin.ml@jages.net. All JAGES datasets have ethical or legal restrictions for public deposition because of the inclusion of sensitive information from participants. Following the regulations of local governments, which cooperated in the survey, the JAGES data management committee has imposed data restrictions.

## Abbreviations

CIs: Confidence intervals
HR: Hazard ratios
JAGES: Japan Gerontological Evaluation Study
